# ORF2 of hepatitis E virus: genetic and antigenic variation across genotypes and implications for immunity, diagnostics and vaccine development

**DOI:** 10.1099/jgv.0.002342

**Published:** 2026-10-08

**Authors:** Sherif A. El-Kafrawy, Sigit S. Putra, Ayman T. Abbas, Umama A. Abdel-dayem, Thamir A. Alandijany, Hisham N. Altayb, Esam I. Azhar

**Affiliations:** 1 Special Infectious Agents Unit-BSL3, King Fahd Medical Research Center, King Abdulaziz University, Jeddah, Saudi Arabia; 2 Department of Medical Laboratory Sciences, Faculty of Applied Medical Sciences, King Abdulaziz University, Jeddah, Saudi Arabia; 3 Animal Facility Unit, King Fahd Medical Research Center, King Abdulaziz University, Jeddah 21589, Saudi Arabia; 4 Biochemistry Department, Faculty of Sciences, King Abdulaziz University, Jeddah, 21589, Saudi Arabia

**Keywords:** antigenic diversity, cross-genotype immunity, genetic variation, hepatitis E virus, ORF2 capsid protein, ORF2 isoforms, pan-genotype diagnostics, vaccine development

## Abstract

Hepatitis E virus (HEV) causes over 19 million infections annually, yet the molecular determinants governing its diverse clinical phenotypes and cross-genotype immunity remain incompletely synthesized. The ORF2 capsid protein is the sole structural component of the virion, the primary neutralizing antibody target and the basis for all commercial diagnostics and the only licensed vaccine (Hecolin®). While current literature often treats HEV as a monolithic antigenic entity, ORF2 sequences diverge by 15–25% across the eight recognized genotypes (GT1–GT8), with critical substitutions concentrated in the protruding (P) domain neutralizing epitopes. This comprehensive review integrates recent structural, virological and clinical data to evaluate the multidimensional impact of ORF2 variability. We highlight how the differential expression of three ORF2 isoforms (infectious ORF2i, secreted decoy ORF2g and cleaved ORF2c) complicates immune recognition. Furthermore, we critically analyse the functional consequences of P domain divergence, which results in 2–4-fold reduced cross-genotype neutralization by GT1-derived vaccines against the globally emerging GT3 and drives false-negative rates of 12–30% in commercial serological assays. Finally, we map ORF2 genetic signatures to distinct clinical phenotypes, including GT1-associated pregnancy mortality and GT3-driven chronicity in transplant recipients. By synthesizing these fragmented domains, this review identifies critical knowledge gaps, particularly concerning the zoonotic emergence of GT7/GT8 and provides a prioritized research roadmap for the development of true pan-genotype vaccines and universal diagnostic algorithms.

## Data availability

 No new data were generated in this review. All cited studies are publicly available through the referenced publications. The phylogenetic analysis presented in Fig. 2 was performed on publicly available ORF2 nucleotide sequences retrieved from GenBank on 15 July 2026 under the following accession numbers: GT1, AF444002, M80581 and JF443721; GT2, M74506 and KX578717; GT3, AF060669, JQ679013 and FJ705359; GT4, AB197674, GU119960 and HQ634346; GT5, AB573435; GT5/GT6, AB602441; GT6, LC789535; GT7, KJ496143, KJ496144 and KT818608; GT8, KX387865, KX387866 and KX387867. Rat hepatitis E virus (*Rocahepevirus ratti*) accession MK050105 was used as the outgroup. The alignment and the resulting tree file are available from the corresponding author upon reasonable request.

## Introduction

Hepatitis E virus (HEV) represents a major global public health challenge, responsible for an estimated 19.47 million cases of acute hepatitis and 3,450 deaths annually worldwide [1, 2]. ∼939 million individuals globally have been exposed to HEV, with 15–110 million experiencing recent or ongoing infection [2–4]. The epidemiology exhibits striking geographic heterogeneity: in low- and middle-income countries of Asia, Africa and the Middle East, genotypes 1 and 2 (GT1, GT2) cause large-scale waterborne outbreaks with case fatality rates reaching 15–30% in pregnant women [3–5]. In contrast, high-income countries experience predominantly sporadic autochthonous infections caused by zoonotic GTs 3 and 4 (GT3, GT4), transmitted through consumption of undercooked pork, wild boar and deer meat. Nationwide surveys of retail pork products continue to identify HEV RNA in commercially available meat, underscoring the ongoing foodborne risk in high-income settings [6–10].

While most infections resolve spontaneously, immunocompromised patients, particularly solid organ transplant (SOT) recipients, face a 60–70% risk of developing chronic hepatitis E, which can progress rapidly to cirrhosis [11–15]. Additionally, extrahepatic manifestations, including neurological complications [Guillain–Barré syndrome (GBS), neuralgic amyotrophy (NA)], haematological abnormalities and renal injury have been increasingly recognized and have been systematically catalogued across multiple organ systems [12–14, 16].

HEV exhibits remarkable genetic diversity, currently classified into at least eight genotypes (GT1-GT8) within the species *Paslahepevirus balayani* (genus *Paslahepevirus*, family *Hepeviridae*), formerly designated *Orthohepevirus* A [17–19]. GT1 and GT2 are exclusively human pathogens endemic to regions with inadequate sanitation [20–23]. GT3 and GT4 exhibit broad mammalian host ranges, circulating in domestic pigs, wild boar, deer and other species with frequent zoonotic transmission [24, 25]. Recently, GT7 and GT8 were discovered in dromedary camels with confirmed human infections, including a documented case of acute hepatitis in a liver transplant recipient (Table 1) [26, 27].

**Table 1.** Virological and epidemiological characteristics of HEV genotypes GT1-GT8

Genotype	Primary host range	Main geographic distribution	Dominant transmission route	Approximate ORF2 divergence vs. GT1 (aa level)
GT1	Humans only [1, 2]	South/Southeast Asia, parts of Africa, Middle East, Mexico [1, 2]	Waterborne, large faecal-oral outbreaks via contaminated drinking water; person-to-person less common [1–4]	Reference genotype; by definition 0%
GT2	Humans only [1, 2]	West/Central Africa and Mexico; circulation less well characterized, fewer contemporary sequences [1–3]	Waterborne faecal-oral outbreaks, similar to GT1 [1, 4]	~15–20% aa divergence in ORF2 vs. GT1 [2, 8, 9]
GT3	Zoonotic: pigs, wild boar, deer, rabbits, other mammals, humans [1, 2, 11]	Worldwide; sporadic autochthonous cases in Europe, North America, parts of Asia and Oceania [1, 2, 11]	Food-borne zoonotic (undercooked pork, wild game), transfusion, organ transplantation; occasional water/environmental exposure [4, 11, 15]	~20–25% ORF2 aa divergence vs. GT1; ~80–84% identity to GT4 in ORF2 [2, 8, 9]
GT4	Zoonotic: pigs, wild boar, other mammals, humans [1, 2, 11]	East and Southeast Asia (China, Japan, Taiwan, parts of South Asia); sporadic reports elsewhere [1–3, 11]	Food-borne zoonotic (pork, wild boar), possibly environmental; transfusion/iatrogenic transmission also reported [4, 13, 14]	Similar ORF2 divergence to GT3 vs. GT1 (≈20–25%); GT3-GT4 ORF2 identities around 80–84% [2, 8, 9]
GT5	Zoonotic: wild boar, probably other suids; human infection uncertain/rare [2, 8, 11]	Mainly reported from Japan and limited areas where wild boar are sampled; very sparse sequence data [2, 8, 11]	Presumed food-borne zoonotic via wild game; no clear human transmission pattern established [2, 8]	15–30% inter-genotype ORF2 divergence vs. GT1; precise percentage depends on strain comparisons [2, 8, 9]
GT6	Zoonotic: wild boar and related suids; rare human infection [2, 8, 11]	East Asia (especially Japan and parts of China); limited sequence sampling [2, 8, 11]	Likely food-borne zoonotic via wild game/pork; human transmission pattern poorly defined [2, 8]	ORF2 divergence comparable to GT5 vs. GT1 (≈20–30% range) [2, 8, 9]
GT7	Zoonotic: dromedary camels and humans [2, 6, 11]	Arabian Peninsula and regions where dromedaries are common; documented human case in a liver-transplant recipient consuming camel products [2, 6, 11]	Zoonotic food-borne route (raw or undercooked camel meat/milk); possibly transfusion or transplantation in chronically infected hosts [2, 6, 11]	∼20–25% ORF2 divergence vs. GT1; 75–82% nucleotide identity across ORF2 compared with GT1-4 [2, 8, 9]
GT8	Zoonotic: Bactrian camels; human infection documented but extremely rare [2, 11]	Regions where Bactrian camels are raised (Central and East Asia); very few sequences and case reports [2, 11]	Presumed zoonotic transmission via camel products; specific routes not well defined [2, 11]	Similar to GT7: ~20–25% ORF2 divergence vs. GT1; grouped with camel-associated strains showing 75–82% ORF2 nucleotide identity to GT1-4 [2, 8, 9]

* Virological and epidemiological overview of the eight recognized genotypes (GT1-GT8) of the species *P. balayani *(formerly *Orthohepevirus* A). Columns include primary host range, geographic distribution, dominant transmission route and ORF2 aa divergence relative to GT1. GT1 and GT2 are obligate human pathogens; GT3 and GT4 are zoonotic with broad mammalian reservoir hosts; GT5 and GT6 infect wild boar with no confirmed human disease; GT7 and GT8 have been identified in dromedary and Bactrian camels, respectively, with documented human spillover infections. Abbreviations: GT, genotype; HEV, hepatitis E virus.

The ORF2 gene encodes the viral capsid protein responsible for virion assembly, host cell interactions and immune recognition [28, 29]. As the sole structural component of the viral capsid, ORF2 (∼660 aas in length, 72 kDa) forms icosahedral shells protecting viral RNA and mediating receptor attachment [30, 31]. High-resolution structures reveal three domains: the shell (S) domain forming the internal scaffold, the middle (M) domain contributing to stability and the protruding (P) domain extending outward to engage receptors and antibodies (Fig. 1) [32, 33].

**Fig. 1.**
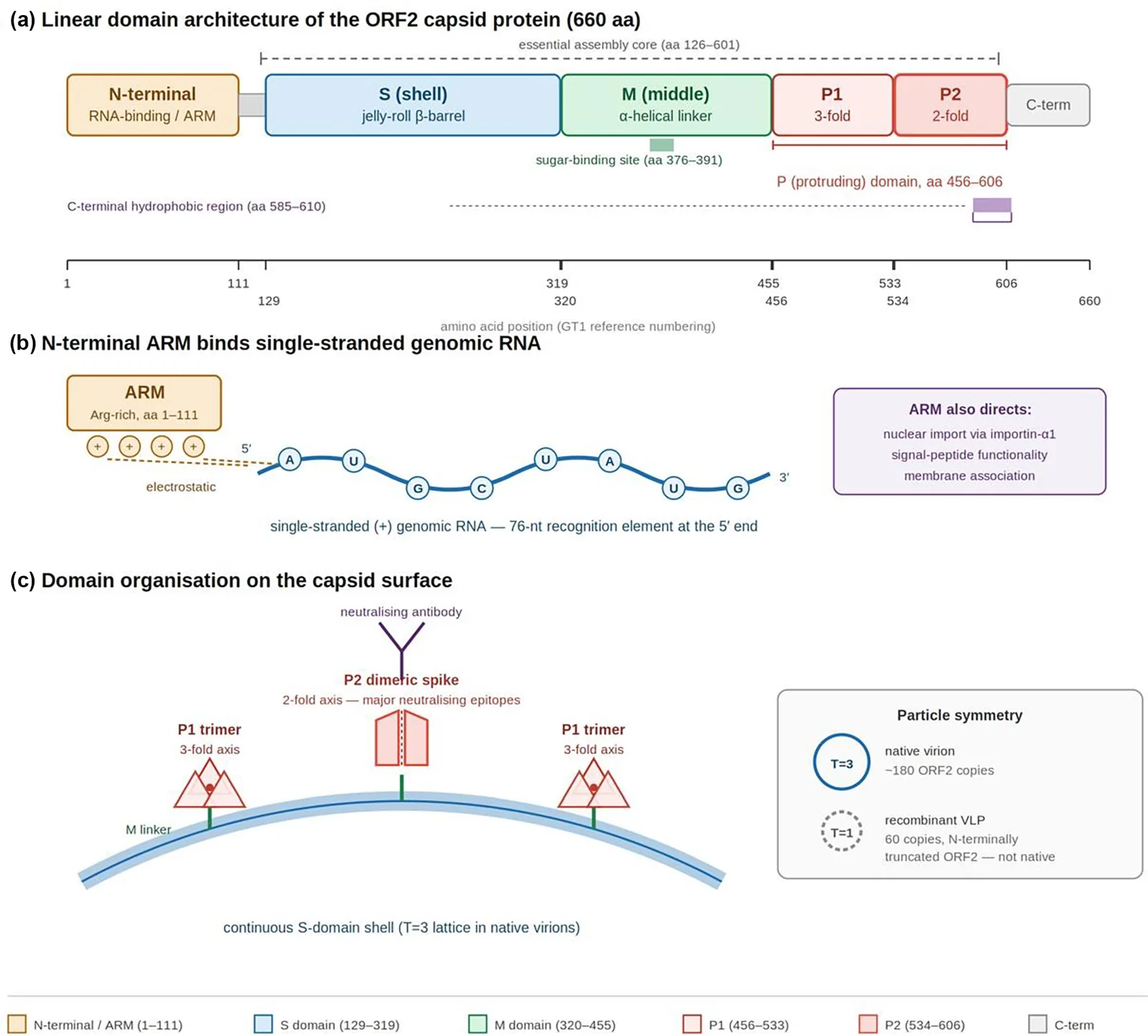
Structural topography and functional domains of the HEV ORF2 capsid protein. (**a**) Linear domain architecture of the ORF2 protein (660 aas in length), drawn to scale against an aa ruler. The N-terminal region (residues 1–111) contains the RNA-binding domain essential for viral encapsidation, including a conserved 76-nucleotide recognition sequence at the 5′ end of the HEV genome. The essential assembly core (residues 126–601) represents the minimal region required for virus-like particle (VLP) formation. Three structural domains are defined: the S domain (~residues 129–319), characterized by a jelly-roll β-barrel fold forming the continuous internal capsid shell; the M domain (~residues 320–455), an α-helix- and loop-rich structural linker connecting the S and P domains and providing conformational flexibility for capsid assembly and disassembly; and the P domain (~residues 456–606), which projects outward from the capsid surface and subdivides into the P1 subdomain (~456–533), a β-barrel containing a sugar-binding site (residues 376–391) involved in receptor attachment and the P2 subdomain (~534–606), which forms dimeric spike-like protrusions harbouring the major neutralizing epitopes. The C-terminal hydrophobic region (residues 585–610) is critical for homo-oligomerization and proper capsid biogenesis. The Arginine-Rich Motif (ARM) in the N-terminal region regulates nuclear translocation via Importin-α1 interaction, signal peptide functionality and membrane association. Note: domain boundaries are approximate and may vary slightly between genotypes and among published crystal structures; residue numbering follows the GT1 reference sequence. (**b**) The N-terminal ARM engages single-stranded, positive-sense genomic RNA through electrostatic contacts between arginine residues and the phosphate backbone, recognizing a 76-nucleotide element at the 5′ end of the genome; the nucleic acid is ssRNA, not DNA. (**c**) Organization of the domains on the capsid surface: the S domain forms the continuous shell, the M domain acts as a linker, the P1 subdomain assembles into trimeric protrusions at the threefold axes and the P2 subdomain forms dimeric spikes at the twofold axes that carry the major neutralizing epitopes and are the principal target of neutralizing antibodies. The inset distinguishes the T=3 icosahedral symmetry of native virions (∼180 ORF2 copies) from the T=1 symmetry (60 copies) observed only in recombinant VLPs assembled from N-terminally truncated ORF2. Note: domain boundaries are approximate and may vary slightly between genotypes and among published crystal structures; residue numbering follows the GT1 reference sequence. Domain boundaries are approximate and vary slightly between genotypes and published structures; residue numbering follows the GT1 reference sequence.

ORF2 exists in at least three functionally distinct isoforms: infectious ORF2 (ORF2i) is non-glycosylated and assembles into capsids; glycosylated ORF2 (ORF2g) is secreted into the bloodstream and may function as an immune decoy; and cleaved ORF2c of uncertain function [34]. ORF2 serves as the primary antigenic target during infection, with neutralizing antibodies predominantly recognizing conformational epitopes in the P domain [35]. The only licensed vaccine, Hecolin® (approved in China but not WHO-prequalified or EMA/FDA-approved), consists of recombinant GT1 ORF2 protein (aas 368–606) demonstrating 96.1% efficacy against GT1 [36]. All commercial serological assays employ recombinant ORF2 antigens [37].

Despite ORF2’s central importance, critical gaps exist regarding how genetic and antigenic variation across genotypes impacts viral fitness, immune recognition, vaccine efficacy and diagnostic performance [38, 39]. Phylogenetic analyses show ORF2 nucleotide sequences diverge by 15–25% between major genotypes, with aa differences concentrated in the P domain where neutralizing epitopes reside [39]. This variability alters antibody recognition: neutralization studies demonstrate reduced cross-genotype neutralization [40]; diagnostic assays show variable sensitivity across genotypes, with some GT3/GT4 infections missed by GT1-based assays [41]; and chronic infection patterns suggest genotype-specific immune evasion mechanisms [15].

This review provides a comprehensive, critical synthesis of current knowledge regarding genetic and antigenic variation within HEV ORF2 across all recognized genotypes, evaluating functional, immunological and clinical implications. We discuss: (i) patterns and drivers of nucleotide and aa variation; (ii) structural and functional consequences for capsid assembly and receptor interactions; (iii) impact of epitope variability on immune recognition and cross-genotype immunity; (iv) implications for vaccine design; (v) effects on serological diagnostic performance and (vi) clinical correlates of ORF2 variability. Understanding ORF2 genetic diversity is fundamental to achieving universal vaccines, robust diagnostics and effective clinical management across diverse epidemiological settings.

## Structural and functional overview of HEV ORF2

### ORF2 genomic organization and expression

The ORF2 gene occupies ∼1,980 nucleotides positioned immediately downstream of ORF1, partially overlapping with ORF3 [42]. Transcription occurs from a bicistronic subgenomic RNA (~2.2 kb), allowing coordinated expression of ORF2 and ORF3. Translation involves two functionally distinct initiation sites within the ORF2 coding region: the first in-frame AUG (Met-1) directs synthesis of the signal-peptide-bearing precursor that enters the secretory pathway and gives rise to the glycosylated ORF2g and cleaved ORF2c forms, whereas an internal downstream AUG generates the non-glycosylated, cytosolic ORF2i form that is incorporated into infectious particles [43]. ORF2 comprises 660 aas in most genotypes, though minor length variations exist [17].

### ORF2 protein isoforms and functional diversity

ORF2 produces three functionally distinct glycosylated and non-glycosylated isoforms (namely ORF2i, ORF2g and ORF2c) [28, 29]. The glycosylation status of these isoforms affects protein interactions, as the phosphorylated form of ORF3 preferentially interacts with the non-glycosylated form of ORF2. ORF2i (72 kDa) is non-glycosylated, translated from an internal AUG codon (methionine-26) and constitutes the authentic viral capsid. Native virions display T=3 icosahedral symmetry built from ∼180 ORF2 copies, whereas the smaller 60-copy T=1 arrangement is a property of recombinant virus-like particles (VLPs) assembled from N-terminally truncated ORF2 rather than of the native particle [31, 36, 44, 45]. ORF2g (88 kDa) is translated from the first AUG, contains an N-terminal signal peptide directing ER entry and undergoes *N*-glycosylation at three conserved residues (N137, N310, N562) [45]. ORF2g is secreted in large quantities but does not incorporate into virions, instead functioning as an immunological decoy binding circulating antibodies [46, 47]. Glomerular deposits of ORF2 protein-antibody complexes have been demonstrated in patients with hepatitis E-associated glomerulonephritis and reduced immune status, providing direct histological evidence that circulating ORF2 forms pathogenic immune complexes [33, 35, 48]. ORF2c (~53–60 kDa) represents a cleaved form of uncertain biogenesis and function [18, 29, 33, 35].

These isoforms have critical implications: differential ORF2i:ORF2g ratios may influence virion assembly vs. immune evasion [45, 46]; diagnostic assays employing glycosylated vs. non-glycosylated antigens detect antibodies with different specificities [47]; and bacterially-expressed vaccines (like Hecolin®) predominantly mimic ORF2i, potentially not eliciting optimal antibodies against ORF2g [47].

### Three-dimensional structure and domain architecture

Recombinant ORF2 constructs with N-terminal truncations self-assemble into T=1 particles. The first T=1 structure was solved at 22 Å resolution using cryo-electron microscopy, demonstrating that a 50 kDa truncated ORF2 protein forms hollow T=1 particles with protruding dimers [49]. T=1 VLPs were further characterized as RNA-free structures [49]. In contrast, full-length or near-full-length ORF2 produces virion-sized T=3 capsids. Direct comparison of both forms showed that the virion-sized capsid demonstrates a T=3 icosahedral lattice and contains an RNA fragment, in contrast to the RNA-free T=1 capsid [49]. Native T=3 capsids contain flat dimers with less curvature than T=1 VLPs, suggesting RNA binding drives assembly of the native T=3 form. Thus, both symmetries are authentic ORF2 structures with distinct biological roles.

Consistent with the RNA-dependent assembly of native T=3 capsids described above, the N-terminal 111 aas of ORF2 contain RNA-binding activity that specifically binds a 76-nucleotide region at the 5′ end of the HEV genome, including a conserved 51-nucleotide sequence. This interaction likely plays a role in viral encapsidation. The essential elements for VLP assembly were narrowed to aas 126–601, with earlier studies showing that proteins consisting of aas 112–660 or 112–608 could also form VLPs. Each protomer contains three domains [18]: the S domain (aas~129–319) forms the interior scaffold with a jelly-roll β-barrel fold, creating a continuous internal shell through inter-subunit interactions [31]. The M domain (~320–455) serves as a structural linker connecting S to P domain through α-helices and loops, allowing conformational flexibility for assembly/disassembly [28, 30, 36, 49]. The P domain (~456–606) projects outward and subdivides into P1 (456–533) and P2 (534–606) subdomains, forming spike-like protrusions with 30 dimers on the capsid surface [30, 32]. Major neutralizing epitopes localize to P2, particularly surface-exposed loops at dimer apexes and flanks [30, 44, 47]. The C-terminal hydrophobic region (585–610) is critical for capsid biogenesis. Deletion of this region resulted in reduced homo-oligomerization and aberrant protein folding. The Arginine-Rich Motif (ARM) in the N-terminal region controls multiple ORF2 functions, including nuclear translocation, signal peptide functionality and membrane association [50]. The ARM interacts with Importin-α1 to facilitate nuclear import and regulates the partitioning and stoichiometry of ORF2 between nuclear, cytosolic and reticular pathways (Fig. 1).

### Receptor binding and capsid assembly

Heparan sulphate proteoglycans (HSPGs), particularly syndecan-1, serve as critical attachment factors, with binding localized to the P domain showing specificity for 6-O-sulfated moieties [37]. Additional receptors include integrin α3 and potentially asialoglycoprotein receptor, with genotype-specific variations in receptor usage contributing to differences in cell tropism and host range [28, 29, 33, 38]. Both the P1 and P2 domains contain potential polysaccharide-binding sites involved in receptor binding. The P1 domain has a conserved sequence (376–391) for potential sugar binding, whereas the P2 domain is structurally similar to receptor-binding domains of other viruses. Sugar binding to the P1 domain may lead to conformational changes that facilitate capsid disassembly and cell entry. Receptor binding to P1 may destabilize the capsid protein trimer, leading to membrane penetration and genome release [19, 29, 31].

A further layer of complexity arises from the fact that HEV circulates in two structurally distinct virion forms that engage the host cell by different routes. Virions shed in faeces and bile are non-enveloped (naked) particles with ORF2 directly exposed on the surface, whereas particles circulating in blood and produced in cell culture are quasi-enveloped, cloaked in a host-derived lipid membrane in which ORF2 is largely masked [51]. This distinction has direct mechanistic consequences: naked virions attach through direct ORF2-HSPG interaction and are highly susceptible to neutralizing antibodies, whereas quasi-enveloped virions are relatively resistant to antibody-mediated neutralization and are thought to enter cells by a lipid-dependent, clathrin-mediated endocytic route requiring endosomal degradation of the lipid coat before ORF2 can engage its receptors. Because ORF2 antigenic variation can only be sensed by the immune system when the capsid is accessible, the naked form is the principal target of vaccine-induced and therapeutic antibodies, while the quasi-enveloped form contributes to persistence in the immunocompromised host. Genotype-dependent differences in the efficiency of quasi-envelopment and in lipid-coat composition remain largely uncharacterized and represent an important variable when interpreting cross-genotype neutralization data.

ORF2 undergoes concentration-dependent self-assembly driven by protein-protein interactions, with dimeric P domain intermediates serving as building blocks [28, 29, 33, 38]. The virion-sized capsid demonstrates a T=3 icosahedral lattice, whereas smaller T=1 capsids can also form. Both capsid types share a common decameric organization, and *in vitro* assembly demonstrated that HEV capsid protein has the intrinsic ability to form decameric intermediates [28, 49]. The assembly of the T=3 capsid involves two types of dimers: flat dimers on twofold axes and inwardly bent dimers on quasi-twofold axes. Assembly likely proceeds through the formation of trimers of dimers that oligomerize at specific symmetry axes. The S domain forms the continuous capsid shell and is critical for assembly, as it cannot be closed by P1 and P2 domains alone. The P1 domain forms trimeric protrusions at threefold axes, whereas the P2 domain forms dimeric spikes at twofold axes. Critical assembly residues include tyrosine-288 at fivefold symmetry axes [31, 36]. HEV capsids exhibit remarkable stability resistant to detergents, organic solvents and low pH, though genotype-dependent stability differences may influence environmental persistence and transmission efficiency.

## Genetic variation in ORF2 across HEV genotypes

### Nucleotide diversity and phylogenetic relationships

ORF2 exhibits substantial nucleotide diversity with pairwise sequence identity ranging from 74–92% across complete gene sequences (Table 1) [17, 18, 24, 25]. GT1 and GT2 share 81–83% identity with each other but only 74–79% with zoonotic GT3/GT4 [37]. GT3 and GT4 display 80–84% identity, reflecting closer phylogenetic relationships [27]. Camel-associated GT7/GT8 show 75–82% identity with GT1-GT4 [26]. In the immunologically critical P domain, divergence is more pronounced with nucleotide identities as low as 70–75% between distant genotypes [52]. The phylogenetic relationships among ORF2 sequences from all eight genotypes are shown in Fig. 2.

**Fig. 2.**
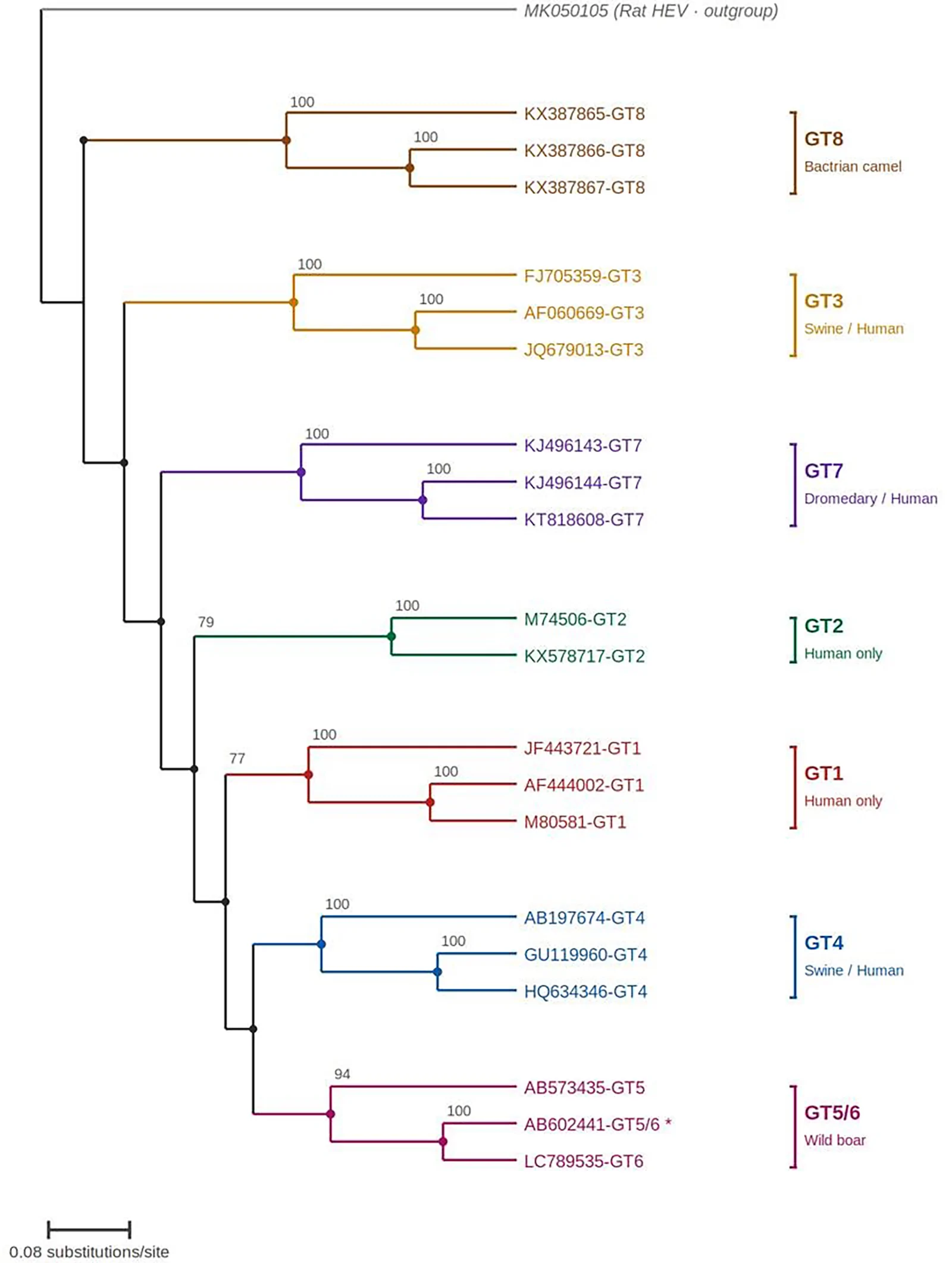
Phylogenetic analysis of HEV ORF2 capsid protein sequences across all eight recognized genotypes of the species *P. balayani*, formerly *Orthohepevirus* A. A maximum-likelihood phylogenetic tree was constructed based on nucleotide sequences of the complete ORF2 coding region (~1,980 nt) from representative strains of HEV (HEV) genotypes 1 through 8. Sequences were retrieved from GenBank (National Center for Biotechnology Information) on 15 July 2026 and aligned using muscle; accession numbers are given at each tip and are listed in full, with their retrieval date, in the Data availability statement. The tree was inferred using mega11 with the GTR+G substitution model, selected by the best-fit model criterion. Branch support was assessed by 1,000 bootstrap replicates; values ≥70% are shown at nodes. The scale bar represents 0.08 nucleotide substitutions per site. Rat HEV (*Rocahepevirus ratti*; GenBank: MK050105) was used as the outgroup. Each sequence is labelled with its GenBank accession number and genotype assignment. Genotypes are colour-coded as follows: GT1, red; GT2, green; GT3, orange; GT4, dark blue; GT5/GT6, dark brown; GT7, purple; GT8, dark red. Accession AB602441 is labelled GT5/6 owing to conflicting genotype assignments in the literature; classification follows Smith *et al*. [17]. The topology of the ORF2-based tree, particularly the basal positioning of the GT7/GT8 camel-associated clade and the clustering of GT1 and GT2 as sister groups, is consistent with the known ORF2-specific evolutionary pressures discussed in Section 3. Intra-genotypic diversity is represented by two to three sequences per genotype where available; GT5 and GT6 are represented by the only complete genome sequences currently deposited in GenBank, reflecting the limited sequence data for these wild boar-associated genotypes. Bootstrap support values ≥70% are shown at nodes. * AB602441 has ambiguous GT5/GT6 assignment; classification follows Smith *et al*. [17].

Intra-genotype diversity of the ORF2 gene varies substantially. All identity values reported in this section refer to ORF2 nucleotide sequences unless otherwise stated. GT1 is the most conserved genotype, with isolates within a subtype sharing more than 95% ORF2 nucleotide identity, consistent with its human-restricted host range; by contrast, GT3 is markedly more heterogeneous, with intra-genotype identity ranging from 84–95%, necessitating subdivision into 11 subtypes (3a–3j, 3ra) [17, 20, 53]. GT4 similarly shows substantial variation (minimum 86.4% identity) with eight subtypes (4a–4h) [8, 22, 54]. Evolutionary rates differ: GT3 evolves at 1.21–1.51×10⁻³ substitutions/site/year vs. 0.85–1.15×10⁻³ for GT1, with major genotypes diverging 536–1,200 years ago [26].

### Selection pressure and codon-specific evolution

Selection pressure acting on a coding sequence is conventionally summarized by the dN/dS ratio, the ratio of non-synonymous (aa-changing) to synonymous (silent) substitutions per site; values below 1 indicate purifying (negative) selection removing deleterious changes, values close to 1 indicate neutral evolution and values above 1 indicate positive (diversifying) selection favouring aa change. Overall dN/dS ratios for ORF2 range from 0.15 to 0.35, indicating that the gene as a whole is under purifying selection [25, 55]. However, regional heterogeneity exists: S and M domains show strong purification (dN/dS ≈0.08–0.18), whereas the P domain exhibits relaxed constraint (0.25–0.45) with several codons approaching dN/dS ≥1.0, suggesting episodic positive selection [30, 32, 44, 47, 55]. Site-specific analyses identified 15–23 positively selected positions, >70% mapping to P2 subdomain (534–606), coinciding with neutralizing epitopes [30, 44, 47, 55]. Specific examples include positions 477, 497, 578 and 602, where substitutions alter antibody binding [30, 55].

Selection patterns differ among genotypes: GT3/GT4 show episodic positive selection at receptor binding and immune recognition sites reflecting multi-host adaptation, whereas GT1 displays uniform purifying selection consistent with human restriction [25, 53, 55]. Host-specific aa substitutions correlate with human vs. swine origin, indicating host immune pressures drive adaptive evolution [25, 55]. Codon usage bias analysis reveals natural selection dominates (95–98% of variation), with optimal codons varying among genotypes correlating with host tRNA abundance [25, 55].

### Genotype-specific signatures and recombination

Signature pattern analysis identified 127 nucleotide positions differentiating major genotypes with >95% specificity [17, 24, 37]. Examples include GT1-specific cytosine at position 1,554 vs. GT3 thymine and a GT4-specific 6-nucleotide motif (ATGCCA) at positions 1,689–1,694 [52, 54]. At the aa level, position 477 exemplifies genotype signatures: GT1/GT2 encode arginine (R477), whereas GT3/GT4 encode lysine (K477), altering local electrostatics and epitope presentation [30, 45].

Although initially considered rare, recombination occurs in ORF2. The Xinjiang-1 strain is a double recombinant between GT1 and GT3, with a breakpoint at nucleotide ~6,500 within ORF2, near the M/P domain boundary [22, 53]. Additional recombinants include GT3/rabbit HEV and intra-GT4 recombinants [56]. Functional consequences could include generating novel epitope combinations enabling immune escape or facilitating host adaptation [21, 23, 57].

### Evolutionary drivers

Multiple forces generate ORF2 diversity [57]: the HEV RNA-dependent RNA polymerase lacks proofreading exonuclease activity, yielding error rates of 10⁻⁴−10⁻⁵ per nucleotide with transition bias [25]. Host immune selection, particularly neutralizing antibodies, drives adaptive evolution concentrated in P domain epitopes [40, 47, 55]. Zoonotic transmission between humans and swine creates divergent selection pressures with host-specific adaptation signatures [21, 23, 25, 53, 55]. Geographic isolation maintains genetic structure, generating region-specific signatures with direct consequences for diagnostic and vaccine performance. The clearest examples are the restriction of GT1 to South Asia, Africa and the Middle East and of GT2 to Mexico and West Africa, where limited animal reservoirs and human-to-human faecal-oral transmission sustain locally distinct lineages; the subdivision of GT3 into the European-dominant 3e/3f/3c subtypes vs. the North American 3a/3b lineages; the confinement of GT4 largely to East and Southeast Asia; and the geographic restriction of GT5 and GT6 to Japanese wild boar and of GT7 and GT8 to camel populations of the Arabian Peninsula and northwestern China, respectively. Because commercial serological assays and the licensed vaccine are both derived from GT1 antigens, these regional signatures translate directly into the reduced assay sensitivity and uncertain vaccine coverage described in Section 4 [27, 34, 54]. This evolutionary complexity generates substantial ORF2 diversity, challenging universal control strategies.

## Implications of ORF2 variation for HEV vaccines and diagnostics

### Current HEV vaccines and cross-genotype protection

The only licensed HEV vaccine, HEV239 (Hecolin®), has been approved exclusively in China since 2011 and has not received WHO prequalification, EMA authorization or United States FDA approval, substantially limiting its global availability. Hecolin® comprises a recombinant truncated ORF2 protein from GT1 strain (aas 368–606, corresponding to the P domain) expressed in *Escherichia coli* and formulated with aluminium hydroxide adjuvant [33, 35]. Phase III clinical trials in China demonstrated 96.1% efficacy against symptomatic hepatitis E, with protection sustained for at least 4.5 years [58]. However, this efficacy was established exclusively in a GT4-endemic region of China, and no efficacy trials have been conducted in GT1-endemic regions of South Asia or Africa or in GT3-endemic regions of Europe and North America. The absence of WHO prequalification has prevented deployment in low- and middle-income countries where HEV burden is greatest and the lack of EMA/FDA approval limits access in high-income countries where GT3 chronicity in immunocompromised patients represents a growing clinical challenge.

In addition, randomized, double-blind, placebo-controlled trials evaluating recombinant HEV vaccines in endemic regions have consistently demonstrated high protective efficacy. One Phase 2 trial reported vaccine efficacy of 95.5% (95% CI: 85.6–98.6) after completion of a three-dose schedule, with 66 hepatitis E cases in the placebo group compared to 3 in the vaccine group. In an intention-to-treat analysis including all cases after the first dose (87 total cases), vaccine efficacy was 88.5% (95% CI: 77.1–94.2), with 9 cases observed among vaccine recipients [58, 59]. A larger Phase 3 trial, enrolling ∼50,000 participants per group, reported 15 HEV infections in the placebo group and none in the vaccine group during the 12 months following the final dose, corresponding to 100% efficacy after three doses. Both trials demonstrated favourable safety profiles, with adverse events generally mild and self-limiting; injection-site pain was the only event significantly increased among vaccine recipients in the Phase 2 trial (*P*=0.03).

Evidence on the use of Hecolin in women of childbearing age has since been extended by a phase 4, double-blind, cluster-randomized, controlled trial conducted in rural Bangladesh, a GT1-endemic setting [60]. That trial provided the first effectiveness data for the vaccine outside China and in a population in which HEV-associated mortality is highest, and it also examined pregnancy outcomes among women who were inadvertently vaccinated shortly before or during pregnancy. Although the trial did not identify a definitive causal signal, the observed imbalance in miscarriage among vaccinated women has been raised as a potential safety consideration and remains a live question for regulatory authorities evaluating the vaccine for use in pregnancy. Because pregnant women in GT1-endemic regions constitute precisely the group with the greatest potential benefit from vaccination, resolving this uncertainty through adequately powered pregnancy-safety studies is a prerequisite for WHO prequalification and for any recommendation to vaccinate during pregnancy.

The GT1-derived vaccine antigen shows 89–92% aa identity with GT3 and GT4 P domain sequences, with divergence concentrated in surface-exposed loops harbouring neutralizing epitopes [30]. Experimental studies reveal genotype-dependent neutralization: Hecolin®-induced antibodies neutralize GT1 and GT4 efficiently (titres>1 : 160) but show 2–4-fold reduced activity against GT3 and limited data exist for GT7/GT8 [32, 38]. Structural analysis identified specific aa substitutions at positions 477, 497, 578 and 602 within neutralizing epitopes that differ between genotypes and reduce antibody binding by 30–60% (Fig. 3) [44, 47].

**Fig. 3.**
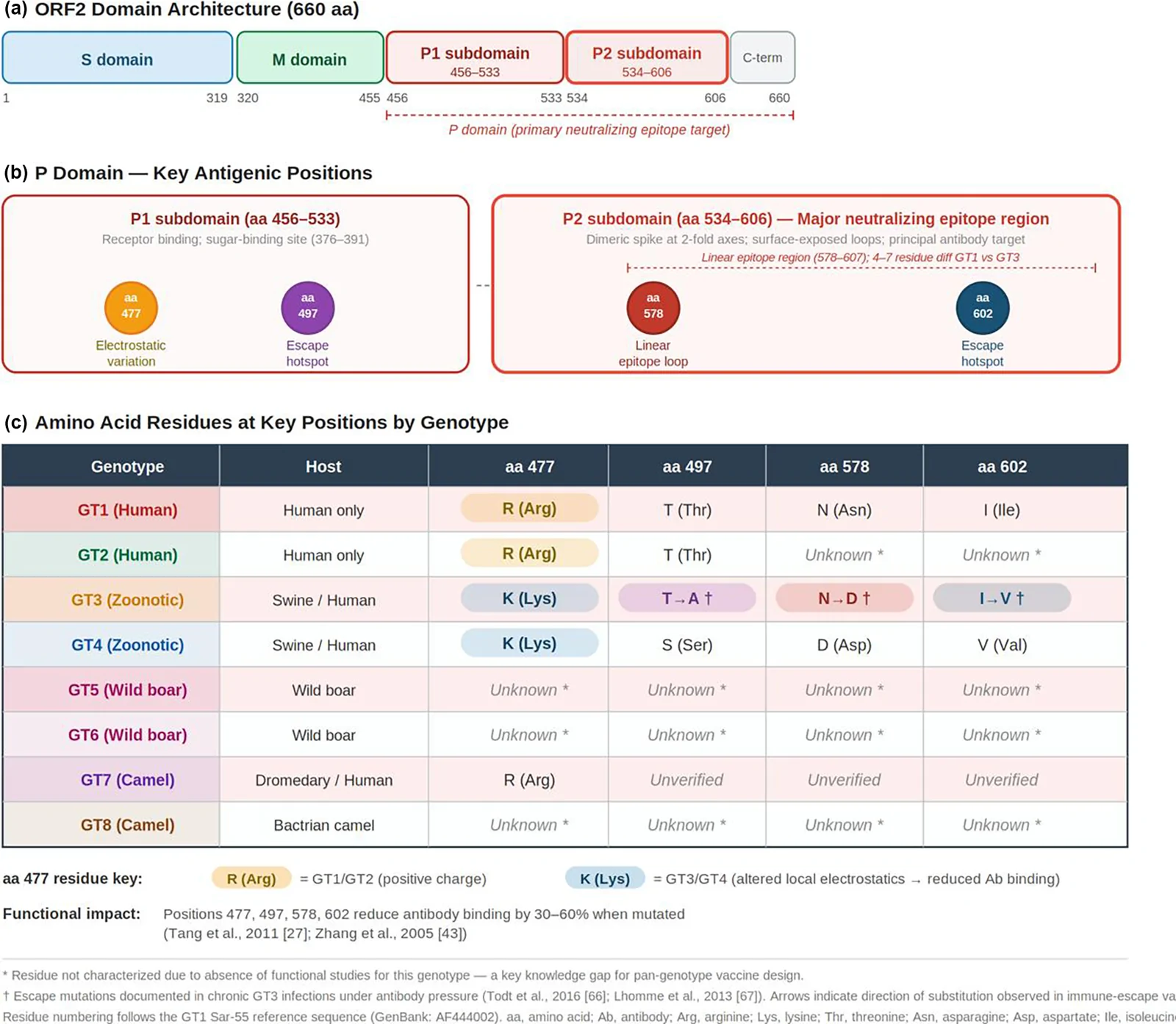
ORF2 P domain antigenic variation at key neutralizing epitope positions across HEV genotypes GT1-GT8. (**a**) Schematic representation of the HEV ORF2 capsid protein (660 aas) drawn to scale, illustrating the three structural domains: the N-terminal region (residues 1–128), which contains the ARM and the RNA-binding activity and does not form part of the assembled shell; the S domain (residues 129–319), which forms the continuous interior capsid scaffold; the M domain (320–455), which serves as a structural linker; and the P domain (456–606), which projects outward from the capsid surface and constitutes the primary target of neutralizing antibodies. The P domain is further subdivided into P1 (456–533), which contains the receptor-binding sugar recognition site (residues 376–391) and P2 (534–606), which forms dimeric spike-like protrusions harbouring the major neutralizing epitopes. (**b**) Expanded schematic of the P domain highlighting the positions of four key aa residues (477, 497, 578, 602) with documented functional consequences for antibody recognition and immune escape. The linear epitope region spanning residues 578–607 is bracketed; this region differs by 4–7 residues between GT1 and GT3, abolishing binding of genotype-specific antibodies. Position 477 lies within P1 and governs local electrostatic charge differences between genotypes. Positions 497, 578 and 602 represent documented immune-escape hotspots in chronic GT3 infections under sustained antibody pressure. (**c**) Genotype comparison table of aa residues at positions 477, 497, 578 and 602 across all eight recognized genotypes of *Orthohepevirus* A. The R/K dimorphism at position 477, arginine (R) in GT1 and GT2 vs. lysine (K) in GT3 and GT4, alters local electrostatics within a neutralizing epitope and is associated with differential antibody-dependent cellular cytotoxicity. Highlighted cells indicate substitutions documented in immune-escape variants emerging during chronic GT3 infection: T→A at position 497, N→D at position 578 and I→V at position 602, each abolishing binding of autologous neutralizing antibodies while maintaining capsid integrity. Residues marked ‘Unknown’ reflect the absence of functional characterization data for GT5, GT6 and GT8, representing a critical gap for pan-genotype vaccine and diagnostic development. GT7 position 477 is inferred from alignment of deposited sequences (GenBank: KJ496143, KJ496144); positions 497, 578 and 602 remain unverified by functional assay. Residue numbering follows the GT1 Sar-55 reference sequence (GenBank: AF444002.1). Genotype colour coding matches Fig. 2. Abbreviations: aa, amino acid; Arg, arginine; Lys, lysine; Thr, threonine; Asn, asparagine; Asp, aspartate; Ile, isoleucine; Val, valine; Ab, antibody. References: Tang *et al*. [30]; Zhang *et al*. [47]; Todt *et al*. [76]; Lhomme *et al*. [77].

Real-world effectiveness data remain limited for heterologous genotypes. Post-marketing surveillance in China (predominantly GT4 circulation) confirms sustained protection, but as noted above, cross-genotype efficacy has not been evaluated in controlled trials outside China [58]. A single case report documented GT3 infection in a Hecolin®-vaccinated individual, though antibody titres were not assessed, precluding definitive conclusions about vaccine failure vs. waning immunity.

### Next-generation vaccine strategies

Addressing genotype diversity requires innovative approaches [17]. Consensus sequence vaccines computationally design antigens maximizing similarity across all genotypes, potentially broadening coverage while maintaining immunogenicity, is an approach yet to be investigated for HEV. In principle, consensus ORF2 P domain antigens could elicit antibodies neutralizing GT1-4 with comparable titres, though this approach has not yet been experimentally validated for HEV, and GT7/8 coverage would require additional assessment.

Multivalent vaccines incorporating P domain antigens from multiple genotypes represent another strategy. Bivalent GT1 +GT3 formulations induced balanced immune responses against both genotypes in preclinical models without immunological interference [61]. However, manufacturing complexity and cost considerations may limit implementation, particularly for resource-limited settings bearing the highest HEV burden [20].

Structure-guided epitope-focused vaccines target conserved conformational epitopes essential for capsid function and, therefore, less tolerant of variation. Monoclonal antibody epitope mapping identified three highly conserved neutralizing epitopes (>95% sequence identity across GT1-8) located at the P domain dimer interface and base [46]. Nanoparticle vaccines displaying only these epitopes elicited broadly neutralizing antibodies in murine and non-human primate models, validating this approach [62].

mRNA and viral vector platforms offer rapid adaptation to emerging genotypes. Self-amplifying RNA platforms encoding ORF2 antigens may offer advantages over protein subunit approaches, including rapid sequence updating and the capacity for multivalent formulations combining sequences from divergent genotypes [62]. Adenoviral vectors expressing ORF2 represent another promising platform, as viral vector vaccines typically elicit both cellular and humoral immunity, which could compensate for reduced antibody cross-reactivity through conserved T-cell epitopes [62].

### Serological diagnostic challenges

Commercial serological assays predominantly employ GT1- or GT3-derived ORF2 antigens (full-length or truncated fragments) for anti-HEV IgM/IgG detection [39]. Comparative evaluations reveal substantial performance variability across genotypes: assays utilizing GT1 antigens demonstrate 95–100% sensitivity for GT1/GT2 infections but only 70–88% for GT3 and 78–92% for GT4, with GT7/GT8 detection largely unvalidated [39, 41]. Conversely, GT3-based assays show reduced sensitivity for GT1 (82–90%), indicating bidirectional epitope mismatch [59].

False-negative results concentrate in specific epidemiological scenarios: GT3 infections in regions employing GT1-based assays (e.g. Asia-Pacific) and GT1 infections in Europe using GT3-optimized assays [63]. Discordant results between assays occur in 12–25% of samples, depending on genotype and assay combinations. Critically, chronic HEV infections in immunocompromised patients, where serology guides treatment decisions, show particularly high false-negative rates (15–30%) with single-genotype assays [64].

The molecular basis involves epitope variability within the ORF2 regions used as antigens. Linear epitopes spanning aas 578–607 differ by 4–7 residues between GT1 and GT3, abolishing binding of genotype-specific antibodies. Conformational epitopes dependent on proper protein folding are additionally affected by expression system (bacterial vs. insect cells) and isoform selection (ORF2i vs. ORF2g), with glycosylation masking epitopes in some assay formats [45].

### Improved diagnostic strategies

Pan-genotype antigen designs incorporating conserved epitopes from multiple genotypes enhance detection breadth [39, 41]. Chimeric antigens combining epitopes from multiple genotypes have shown improved cross-genotype sensitivity in validation studies, with some formulations achieving near-complete detection when GT1 to GT4 ORF2 antigens are used in combination [30, 65]. Similarly, peptide cocktails presenting immunodominant epitopes from divergent genotypes improved detection concordance between reference assays from 78–94% [39, 41].

Dual-antigen algorithms employ parallel testing with GT1 and GT3/4 antigens, interpreting reactivity as positive [39, 41]. While increasing cost and complexity, this approach substantially reduced false negatives in GT3-endemic regions and improved detection of chronic infections in transplant recipients [39, 41]. Dual-antigen confirmation for equivocal results has been increasingly advocated in clinical practice.

Molecular diagnostics complement serology, with RT-PCR targeting conserved ORF2 regions enabling genotype-independent detection of viral RNA [66]. Next-generation sequencing provides simultaneous detection and genotyping, important for emerging genotypes and outbreak investigations [18]. However, molecular methods require viremia (present in only 50–70% of acute infections) and specialized infrastructure, limiting applicability in resource-limited settings.

The short and variable duration of viraemia can be partly offset by testing faecal specimens. HEV is shed in stool from shortly before the onset of symptoms, so faecal testing can detect infection earlier in its course than serum-based molecular assays [67]. Reported shedding kinetics differ appreciably between cohorts, however, and stool positivity does not consistently outlast viraemia: in one serial-sampling study faecal excretion and viraemia were both short-lived and neither persisted beyond biochemical resolution of hepatitis [68], and another reported a maximum of 42 days of viraemia against 28 days of faecal shedding [69], whereas prolonged faecal excretion lasting several months has been documented in individual patients [70]. The established diagnostic role of stool testing lies instead in chronic infection, where European Association for the Study of the Liver guidance recommends that HEV RNA be assessed in both serum and stool at the end of ribavirin therapy and that treatment be continued if either compartment remains positive, since persistent faecal shedding predicts relapse [67]. Faecal specimens also have practical advantages for surveillance and outbreak investigation, since collection is non-invasive and the virions shed in stool are non-enveloped and environmentally stable, in contrast to the quasi-enveloped particles circulating in blood [51, 67]. Two caveats limit wider adoption: faecal positivity falls rapidly after presentation, so a single negative stool result late in the illness does not exclude infection [69], and stool-based assays are less widely standardized than plasma assays, so quantitative results are not readily comparable between centres [67]. Cross-genotype primer coverage in ORF2-targeted assays applies equally to faecal and plasma specimens.

Looking forward, standardization initiatives and evidence-based assay selection matched to local genotype epidemiology will be necessary for optimizing diagnostic accuracy globally; the specific research priorities required to achieve this are discussed in Section 6.4.

## Clinical implications of ORF2 variability

### Genotype-specific disease severity

The clinical impact of ORF2 variability is shaped by genotype distribution, which varies markedly across geographic regions: GT3 predominates in Europe (with subtypes 3c, 3f and 3e representing the majority of typed cases), GT3 and GT4 co-circulate in East Asia, and GT1 remains dominant in South Asia and Africa [71, 72]. These geographic patterns are clinically significant because HEV genotypes exhibit markedly different disease phenotypes that correlate with ORF2 sequence variation.

HEV genotypes exhibit markedly different clinical phenotypes, with accumulating evidence suggesting ORF2 sequence variation may contribute to pathogenic differences (Table 2) [6, 7]. GT1 infections are characterized by severe outcomes in specific populations, particularly pregnant women, in whom case fatality rates reach 15–30% during the third trimester, compared to <1% in non-pregnant adults [4]. Fulminant hepatic failure occurs in 1–3% of GT1 cases overall but escalates to 10–20% in pregnant women, with mechanisms involving immune dysregulation and altered hormonal milieu [73]. In endemic regions, GT1 accounts for a substantial proportion of acute liver failure cases [73].

**Table 2.** Clinical and immunological characteristics of HEV genotypes GT1-GT8

Genotype	Typical disease severity (immunocompetent)	Chronicity in immunocompromised (SOT, hematologic, HIV)	Extrahepatic manifestation (neurologic, renal and others)
GT1	Symptomatic acute hepatitis common; high risk of fulminant hepatitis in pregnancy, maternal mortality up to 15–25% in third trimester [1]	Chronic infection essentially not documented; cases considered exceedingly rare [5, 6]	Extrahepatic disease reported but less strongly linked than GT3 (neurologic, hematologic and renal) [2, 7]
GT2	Acute self-limited hepatitis; severe disease and pregnancy-associated mortality reported but fewer data than GT1 [1]	Chronic infection not convincingly documented; if present, extremely rare [5, 6]	Extrahepatic manifestations rarely described; evidence limited by small case numbers [2, 7]
GT3	Often subclinical or mild; can cause acute hepatitis and occasional severe or fulminant disease [5, 7]	Major cause of chronic HEV: chronicity in 50–70% of infected solid-organ transplant recipients without intervention; also in hematologic malignancy and HIV cohorts [5, 6]	Strongly associated with extrahepatic disease, particularly neurologic syndromes (GBS, NA and encephalitis) and renal/hematologic complications [2, 7]
GT4	Acute hepatitis, often more icteric; some series report higher peak ALT and more jaundice than GT3 but overall similar case-fatality in non-pregnant adults [2, 5, 7]	Chronic infection less frequent than GT3 but well documented; considered ‘exceedingly rare’ in some series and ‘occasional’ in others, including a few persistent cases in children and transplant recipients [5, 6]	Extrahepatic manifestations described (neurologic, pancreatic, hematologic and renal), but association appears weaker and less well quantified than for GT3 [2, 7]
GT5	Clinical phenotype in humans essentially unknown; infections so far mainly defined virologically in animals [2, 8]	No confirmed human chronicity data; risk unknown [2, 8]	Extrahepatic manifestations not characterized [2, 8]
GT6	Human disease characteristics not well defined; assumed similar to other zoonotic genotypes when spillover occurs [2, 8]	No systematic data on chronicity; isolated human cases only [2, 8]	Extrahepatic disease not described [2, 8]
GT7	Limited human data; documented acute hepatitis in an immunocompromised liver-transplant recipient, severity comparable to other acute HEV in that context [2, 6]	At least one case of persistent GT7 infection with chronic hepatitis in a transplant recipient has been reported [6]	Extrahepatic manifestations not systemically described [2, 6]
GT8	Human disease phenotype largely undefined; limited data suggest acute hepatitis similar to other genotypes when infection occurs [2, 11]	No clear evidence on chronicity; insufficient human cases [2]	Extrahepatic manifestations not described [2]

Clinical and immunological overview of the eight recognized genotypes (GT1-GT8) of the species *P. balayani *(formerly Orthohepevirus A). Columns include disease severity in immunocompetent hosts, chronicity risk in immunocompromised populations and principal extrahepatic manifestations. Clinical data for GT5, GT6 and GT8 are extremely limited owing to the absence of confirmed human infections (GT5/GT6) or scarcity of documented cases (GT8). Abbreviations: GT, genotype; HEV, hepatitis E virus; SOT, solid organ transplant; GBS, Guillain–Barré syndrome; NA, neuralgic amyotrophy.

GT3 infections typically cause milder acute hepatitis in immunocompetent hosts (mortality <0.5%) but exhibit a high propensity for chronic evolution in immunocompromised patients [11, 15]. Among SOT recipients, GT3 chronicity rates reach 60–70%, significantly exceeding GT1 chronicity rates (20–30%) under comparable immunosuppression [12]. GT3 is disproportionately associated with neurological complications, occurring in 5–15% of infections v s. <2% for GT1/GT4, including GBS, NA and encephalitis [12, 14]. Proposed mechanisms involve molecular mimicry between GT3-specific ORF2 epitopes and neural antigens, though definitive epitope mapping remains incomplete [74].

Notably, European surveillance data further refine GT3 heterogeneity: within GT 3, clade 3efg (often classified as group 1) has been associated with significantly higher peak bilirubin levels (68.2% increase after multivariable adjustment), higher hospitalization rates (up to 79.5%) and increased fatality compared with clades 3abchijklm (group 2), suggesting that intra-genotypic ORF2 variation may influence clinical severity even within GT3 lineages.

GT4 infections demonstrate intermediate severity profiles but distinctive biochemical signatures: peak ALT levels are 1.5–2-fold higher in GT4 than GT3 infections (median 2,800 vs. 1,600 U l^−1^), with correspondingly higher rates of jaundice (85 vs. 60%) [8]. Direct genotype comparisons in Japanese cohorts further support increased biochemical severity of GT4, with significantly higher peak ALT levels (median 3,430 IU l^−1^ vs. 1,052 IU l^−1^ for GT3) and lower prothrombin activity, indicating more pronounced hepatic dysfunction [71]. In immunocompromised patients, GT4 has been reported to cause faster progression to cirrhosis compared to GT3 and higher rates of decompensation [13]. Notably, a high proportion of GT4-infected transplant recipients have been reported to develop persistent infection compared to higher rates of spontaneous clearance in GT3 chronic cases [75].

The molecular basis linking ORF2 variation to disease severity remains incompletely characterized. One hypothesis posits that genotype-specific ORF2 epitope configurations modulate immunopathology: GT1 epitopes may trigger excessive inflammatory responses contributing to fulminant disease, whereas GT3 epitopes facilitate immune evasion enabling chronicity [3]. Comparative studies identified aa position 477 (R in GT1, K in GT3/GT4) within a neutralizing epitope that correlates with differential antibody-dependent cellular cytotoxicity, potentially influencing hepatocyte injury patterns [30].

However, a multi-country European sequence analysis comparing HEV-3 variants in blood donors and clinically apparent hepatitis cases found no consistent association between viral subgenomic variation and disease severity, highlighting that host factors may outweigh viral genetic determinants in certain populations [72].

### Chronicity and viral persistence

Chronic HEV infection, defined as viral RNA persistence >3–6 months, occurs almost exclusively in immunocompromised individuals and exhibits strong genotype dependence [6, 13]. GT3 accounts for >80% of documented chronic cases worldwide, with chronicity rates of 60–70% in transplant recipients vs. 20–30% for GT1 under comparable immunosuppression [40]. GT4 shows intermediate chronicity but faster fibrosis progression [6, 13].

ORF2 epitope variability enables immune evasion mechanisms sustaining persistent infection [76]. Longitudinal sequencing of chronic GT3 infections revealed accumulation of non-synonymous mutations within ORF2 neutralizing epitopes at rates 5–10-fold higher than synonymous mutations (dN/dS=1.8–3.2 for epitope codons vs. 0.12 overall), demonstrating active antibody-driven selection [76]. Specific escape mutations at positions 497 (T→A), 578 (N→D) and 602 (I→V) abolished binding of autologous neutralizing antibodies while maintaining capsid stability. Notably, these escape variants emerge within 6–12 months of infection onset, coinciding with the transition from acute to chronic phase [77]. Indeed, population-based data indicate that approximately one-quarter of immunocompromised individuals in large European cohorts develop chronic infection, further reinforcing the strong genotype-host interaction underlying persistence.

Therapeutic implications are substantial. Ribavirin monotherapy achieves sustained virological response in 75–85% of GT3 chronic infections but only 50–60% for GT4, with resistance associated with mutations in ORF1 polymerase rather than ORF2 [13]. However, ribavirin-refractory cases (15–25%) often harbour ORF2 variants with reduced antibody recognition, suggesting that inadequate immune control, rather than direct antiviral resistance, underlies treatment failure [76]. Combination approaches adding pegylated interferon improved response rates to 85% in transplant-eligible patients. This gain must, however, be weighed against a substantial toxicity burden: pegylated interferon causes influenza-like symptoms, cytopenias, depression and thyroid dysfunction, requires parenteral administration over months and carries a well-recognized risk of precipitating graft rejection, which makes it contraindicated in most kidney, heart and lung transplant recipients and restricts its use largely to liver transplant recipients under close supervision. The reality that the only salvage option for ribavirin-refractory chronic hepatitis E is a poorly tolerated and often contraindicated therapy underscores how narrow the current therapeutic armamentarium is and provides a strong clinical rationale for developing directly acting antivirals, broadly neutralizing antibody products and pan-genotype vaccines capable of preventing chronic infection in this population [78].

Therapeutic antibodies represent emerging treatments. Convalescent plasma from GT3-recovered donors reduced viral loads by 1–3 log_₁₀_ in autologous genotype infections but showed limited efficacy against heterologous genotypes, emphasizing genotype-matching requirements [79]. Monoclonal antibody cocktails targeting conserved ORF2 epitopes demonstrated broad neutralization across GT1-4 *in vitro* (IC_₅₀_ <50 ng ml^−1^) and prevented chronic infection in humanized mouse models when administered early post-exposure [32].

### Extrahepatic manifestations and pregnancy outcomes

Neurological complications occur in 5–10% of HEV infections, predominantly with GT3, including GBS (3–5%), NA (2–4%), encephalitis (1–2%) and myelitis (<1%) [80]. Proposed mechanisms involve molecular mimicry between ORF2 epitopes (particularly aas 578–607) and myelin proteins, triggering cross-reactive autoantibodies [81]. Supporting this, GT3-infected patients with neurological complications showed higher titres of anti-ganglioside antibodies (GM1, GQ1b) than those without [80]. However, direct ORF2 epitope mapping against neural antigens has not confirmed specific cross-reactive sequences.

Additional extrahepatic manifestations include acute pancreatitis (3–5%), glomerulonephritis (2–3%) and haematological abnormalities, including thrombocytopenia (5–8%) and haemolytic anaemia (1–2%) [14]. Recent studies identified ORF2g-antibody, immune complexes deposited in the renal glomeruli of HEV-associated membranoproliferative glomerulonephritis patients, establishing a direct pathogenic link between secreted ORF2 and immune-mediated extrahepatic disease [48].

Pregnancy outcomes differ dramatically by genotype. GT1 causes maternal mortality rates of 15–25% in the third trimester, with fetal loss rates of 30–50% [82]. In contrast, GT3 and GT4 infections during pregnancy show mortality rates <1%, comparable to non-pregnant populations [83]. Vertical transmission occurs in 30–50% of GT1-infected mothers vs. <10% for GT3/GT4 [73]. The molecular basis remains unclear, though hypotheses include GT1-specific placental tropism mediated by ORF2 receptor-binding variants or exaggerated inflammatory responses to GT1 epitopes during pregnancy-associated immune modulation [4, 77]. No specific ORF2 aa substitutions have been definitively linked to adverse pregnancy outcomes, representing a significant knowledge gap requiring case-control studies with deep sequencing.

### Therapeutic antibodies and escape potential

Passive immunotherapy using HEV-specific antibodies is under active investigation [84]. Convalescent plasma therapy demonstrated proof-of-concept efficacy in small case series: three GT3 chronic infections treated with GT3-convalescent plasma achieved viral clearance, whereas two GT4 patients receiving GT1-convalescent plasma showed no response, highlighting the importance of genotype matching [79]. Polyclonal anti-HEV immunoglobulin preparations purified from high-titre donors are in preclinical development [51].

Monoclonal antibody therapeutics offer potential advantages of defined specificity and unlimited supply [46, 47]. The broadly neutralizing antibody 8G12, targeting a conserved conformational epitope at the P domain dimer interface (residues 455–462, 570–580), neutralizes GT1-4 with comparable potency (IC_₅₀_ 10–35 ng ml^−1^) [32]. However, *in vitro* escape mutation studies revealed that prolonged culture with sub-neutralizing 8G12 concentrations selected variants with E479K or D580N substitutions, reducing antibody binding 100-fold while maintaining viral fitness [32]. This suggests monotherapy risks selecting resistant variants, supporting combination approaches targeting multiple epitopes.

Antibody cocktails combining 3–4 non-competing mAbs targeting distinct ORF2 epitopes prevented escape in serial passage experiments and achieved sterilizing immunity in HEV-infected humanized mice [30]. Importantly, GT7 and GT8 showed reduced susceptibility to GT1-derived mAb cocktails (5–15-fold higher IC_₅₀_), necessitating cocktail optimization for emerging genotypes [26]. These data inform clinical trial design, emphasizing multi-antibody regimens with cross-genotype coverage.

Beyond direct neutralization, antibody-dependent effector mechanisms may contribute to control of HEV, but this remains almost entirely unstudied, and we are not aware of any published investigation of Fc-mediated effector function against HEV. In other viral infections, engagement of Fc gamma receptors by antigen-bound IgG mediates antibody-dependent cellular cytotoxicity and antibody-dependent cellular phagocytosis and complement activation contributes to viral clearance, with non-neutralizing antibodies shown to confer protection through these routes [85]. Whether analogous mechanisms operate against HEV is unknown, and two features of HEV biology make the question non-trivial: ORF2 is masked on quasi-enveloped virions circulating in blood, so Fc engagement would presumably be confined to naked particles and to ORF2 displayed on infected hepatocytes; and the abundant secreted ORF2g form binds circulating antibody and might divert Fc engagement away from infectious particles [45, 46]. It is also unknown whether the genotype-specific residues that modulate neutralization, including the R477K dimorphism, influence Fc-mediated effector function, since no study has compared effector activity across genotypes. Serological profiling that measures effector function alongside neutralizing titre would establish whether current potency assays, which score neutralization alone, underestimate protection conferred by non-neutralizing antibodies.

Passive immunotherapy is conceptually most attractive in the population least able to generate its own response. Chronic HEV infection occurs almost exclusively in immunocompromised individuals, and delayed or absent seroconversion is a recognized feature of infection in SOT recipients, in whom chronic infection, accelerated fibrosis and ribavirin failure are concentrated [6, 13, 67]. Current management relies on reduction of immunosuppression, which achieves clearance in approximately one-third of cases, followed by off-label ribavirin [67]; neither option is a targeted antiviral therapy. A standardized, genotype-matched hyperimmune immunoglobulin or a defined monoclonal cocktail administered at diagnosis is, therefore, a reasonable concept, although polyclonal anti-HEV immunoglobulin preparations remain in preclinical development and no such product has entered clinical evaluation [84]. Prophylaxis before transplantation in endemic regions and pre-emptive treatment of viraemic donors or recipients are further scenarios that would merit formal evaluation, but we emphasize that all of these remain untested proposals rather than established options.

Bispecific antibody formats offer, in principle, a means of raising the genetic barrier to escape without the manufacturing complexity of multi-product cocktails. In HIV-1, bispecific constructs combining two independent broadly neutralizing specificities achieved greater breadth and potency than either parental antibody and restricted the emergence of escape variants, establishing the underlying principle [85]. Applied to HEV, a single bispecific engaging two non-overlapping conserved epitopes, for example, the P domain dimer interface targeted by 8G12 together with a conserved P1 surface, would require simultaneous mutation at two independent sites for escape. Formats co-engaging ORF2 and a hepatocyte-surface molecule might additionally improve hepatic delivery. We note, however, that no HEV-specific bispecific construct has been reported, that the conserved epitopes required to build one have not been comprehensively mapped across genotypes and that this, therefore, remains a hypothesis rather than a development candidate.

Whether the escape substitutions identified *in vitro* will emerge clinically requires careful qualification. The E479K and D580N variants selected under sub-neutralizing 8G12 pressure arose in culture, in a system lacking cellular immunity, competing quasispecies and the fitness costs imposed by transmission bottlenecks, and their emergence, therefore, cannot be extrapolated directly to patients [32]. Two observations nonetheless suggest genuine clinical risk. First, the substitutions documented at positions 497, 578 and 602 during chronic GT3 infection arose *in vivo* under endogenous polyclonal antibody pressure, demonstrating that ORF2 epitopes are mutable within a human host [76, 77]. Second, chronically infected immunocompromised patients represent the intended recipients of antibody therapy and simultaneously provide the prolonged, high-titre replication and partial immune pressure that most favour escape [6, 13]. The most likely clinical scenario is, therefore, selection of escape variants during monotherapy in chronically infected transplant recipients rather than during short-course post-exposure prophylaxis in immunocompetent individuals. This argues for deploying combination regimens from the outset in the chronic setting, for sequencing ORF2 at baseline and at virological rebound in any therapeutic antibody trial and for pre-specifying escape surveillance as a trial endpoint.

Among alternative passive immunotherapy platforms, avian IgY antibodies merit consideration for HEV, particularly in the low- and middle-income settings where GT1 and GT2 outbreaks occur. IgY is the avian functional counterpart of mammalian IgG and is transferred to the egg yolk, from which it can be recovered non-invasively in gram quantities per hen per month, at substantially lower cost than mammalian cell culture-derived monoclonal antibodies [70, 86]. Because IgY does not bind mammalian Fc gamma receptors, does not activate mammalian complement and does not cross-react with rheumatoid factor, it avoids several inflammatory and assay-interference liabilities of heterologous mammalian IgG, and its phylogenetic distance from mammalian immunoglobulins can allow recognition of epitopes that are poorly immunogenic in mammals [86, 87]. For an enterically transmitted virus, these properties are of interest, since orally administered IgY acts within the gut lumen, where HEV virions are shed in their naked, non-enveloped form; oral IgY preparations have reduced enteric infection in animal models and in controlled human studies for other faecal-orally transmitted pathogens, including rotavirus [70, 87]. Recognized constraints of the platform are that oral IgY is degraded by gastric acid and pepsin and, therefore, requires enteric protection or encapsulation [88, 89], and that activity would be expected against luminal naked virions rather than the quasi-enveloped particles circulating in blood [51]. We emphasize that these considerations are entirely extrapolated from other pathogens: no anti-HEV IgY preparation has been reported and neither cross-genotype binding nor neutralization of HEV by IgY has been demonstrated. Generating ORF2-specific IgY against antigens from several genotypes and profiling its cross-genotype neutralizing activity would be an inexpensive first step in establishing whether the platform has any role in HEV control.

## Knowledge gaps and future research directions

Despite substantial advances in HEV ORF2 biology, key gaps impede universal vaccine development, pan-genotype diagnostics and optimized therapeutics. Below, we identify the most pressing unanswered questions [1, 2].

### Understudied genotypes and structural biology unknowns

The most fundamental barrier to universal HEV control is the severe knowledge deficit surrounding understudied genotypes. GT2, the least characterized human-pathogenic genotype, is represented by only a single complete genome sequence [17]; its ORF2 diverges by ∼15–20% from GT1 at the aa level [2, 37], yet virtually nothing is known about its neutralizing epitope topology, receptor binding properties, clinical phenotype in immunocompromised hosts, response to existing vaccines or detectability by commercial serological assays. Whether GT2 can cause chronic infection or extrahepatic disease remains entirely undetermined, and no cell culture system supports GT2 replication [17, 27]. GT5 and GT6, identified primarily in wild boar in Japan [9, 17], have no confirmed human infections and no experimental data on ORF2 antigenicity, cross-reactivity with GT1-derived vaccine antibodies or recognition by diagnostic assays [24]. Whether their ORF2 P-domain epitopes are sufficiently conserved to be neutralized by existing antibody responses or whether they would present novel antigenic surfaces upon zoonotic spillover, is unknown. GT7, identified in dromedary camels with at least one confirmed human infection in an immunocompromised transplant recipient [6, 26, 90], has 20–25% ORF2 divergence from GT1-4, yet its ORF2 structure has not been experimentally determined, its susceptibility to Hecolin®-induced antibodies has not been tested in neutralization assays and its potential for chronic infection beyond the single documented case is uncharacterized [26]. GT8, identified in Bactrian camels, shares similar ORF2 divergence with GT7 but has even fewer available sequences, no confirmed human disease phenotype data and no assessment of diagnostic assay cross-reactivity [2, 26].

These genotype-specific gaps are compounded by broader structural biology unknowns. While crystal structures exist for GT1 (PDB: 2ZTN) and GT3 (PDB: 3HAG), no experimentally determined structures are available for GT4, GT7 or GT8, an important gap given that GT4 causes more severe disease than GT3 despite 80–84% sequence identity. Cryo-electron microscopy of these genotypes would reveal whether the 12–18 divergent P2 residues alter the angle of P-domain protrusion and reshape neutralizing epitope loops. Equally unresolved is how the three ORF2 isoforms differ structurally: whether ORF2g glycosylation at N137, N310 and N562 masks key epitopes, where ORF2c cleavage occurs across genotypes, and whether ORF2g-antibody immune complexes directly drive glomerulonephritis or neurological injury through complement activation. The molecular determinants of concentration-dependent capsid self-assembly, and whether small-molecule disruptors could serve as antivirals, also remain unexplored [28, 32].

### Evolutionary dynamics and genotype-phenotype causality

Correlations between ORF2 genotype and clinical phenotype, GT1 pregnancy mortality, GT3 chronicity and GT4-accelerated cirrhosis remain unproven mechanistically. Chimeric virus experiments (GT1 ORF2 in a GT3 backbone and vice versa) and single-substitution mutagenesis at positions 477 (R/K) and 497 (T/S) in humanized mouse models are needed to establish causality. The intense positive selection during chronic infection (dN/dS=1.8–3.2 for epitope codons vs. 0.12 genome-wide) raises further questions: do escape mutations at 497, 578 and 602 pre-exist in quasispecies or arise *de novo* and do they incur fitness costs constraining evolution? Recombination, documented in Xinjiang-1 and GT3/rabbit strains, may be under-detected due to sampling bias and could theoretically generate pandemic variants with chimeric epitope configurations [1, 22].

### Vaccine immunology and cross-protection mechanisms

As described in Section 4.1, Hecolin®-induced antibodies show 2–4-fold reduced neutralization against GT3 despite 89–92% P-domain identity, but the mechanistic basis of this gap remains unresolved. Systematic epitope mapping using sera from vaccinated individuals is needed to determine whether this reflects immunodominance of variable loops, conformational differences introduced by bacterial expression systems or glycosylation-dependent epitope masking absent in ORF2i-based vaccines. Additionally, the durability of cross-genotype protection beyond GT4-endemic populations is unknown, as is whether heterologous booster doses could broaden waning responses. T-cell epitope conservation across genotypes has not been systematically assessed, leaving open the question of whether cellular immunity can compensate for reduced antibody cross-reactivity. Finally, therapeutic vaccination to clear established chronic infection, potentially combining heterologous ORF2 antigens with TLR agonist adjuvants alongside ribavirin, warrants preclinical evaluation but remains entirely unexplored [3, 32, 46].

Control at the animal reservoir represents a complementary strategy that has received little systematic attention. Because GT3 and GT4 infections in high-income countries are overwhelmingly acquired from domestic pigs, wild boar and deer, reducing viral load in the swine reservoir would be expected to lower human exposure through both food-borne and environmental routes and would benefit precisely those immunocompromised patients least likely to respond to their own vaccination [9, 24]. The experimental evidence for porcine immunization is, however, mixed and cautions against assuming that vaccinating pigs would be straightforward. A VLP vaccine based on the GT3 ORF2 p239 fragment protected pigs against homologous GT3 challenge in a dose-dependent manner, with neither viraemia nor faecal shedding detected in animals receiving the higher dose [88]. By contrast, a subsequent challenge study found that neither Hecolin nor a genotype 3-matched p239 construct prevented GT3 infection in pigs [89]: only one of four animals in each group was fully protected, and viral RNA was recovered from bile or liver in three of four animals in both vaccinated groups despite high neutralizing antibody titres, although the genotype-matched construct did prevent faecal shedding in infected animals. Earlier work assessing cross-protection between swine, rat and avian HEV capsid antigens likewise found protection to be incomplete and antigen-dependent [91]. Taken together, these studies establish that seroconversion in pigs does not reliably equate to protection from infection or shedding and that genotype matching of the immunogen matters. Beyond this immunological uncertainty, the obstacles are principally economic and structural. HEV causes no clinically apparent disease in pigs, so producers have no production-related incentive to vaccinate and the cost would need to be justified entirely on public health grounds [89]; the short interval between weaning and slaughter leaves a narrow window for a multi-dose schedule; wild boar and deer reservoirs are not amenable to systematic vaccination and would sustain transmission even if domestic herds were covered [9, 24]; critically, no study has yet demonstrated an effect of porcine vaccination on transmission, as distinct from seroconversion, at herd scale, and we are not aware of any formal cost-effectiveness modelling of the intervention. A One Health evaluation comparing swine vaccination against non-vaccine measures, such as improved biosecurity, slaughterhouse hygiene, targeted testing of liver-containing products and consumer cooking guidance would, therefore, be required before veterinary vaccination could be advocated as policy.

### Diagnostic assay optimization

Although the genotype-dependent sensitivity gaps and improved antigen strategies described in Sections 4.3 and 4.4 have been characterized, several unresolved questions impede further progress. First, systematic mutagenesis studies linking specific ORF2 residue substitutions to loss of antibody recognition in commercial assay formats are lacking, meaning the precise molecular basis of cross-genotype false negatives remains unclear. Second, point-of-care rapid diagnostic tests, needed in resource-limited endemic settings where HEV burden is highest, have been validated almost exclusively against GT1 reference panels, with minimal evaluation against GT3, GT4 or the emerging GT7/GT8 genotypes. Third, while chimeric and cocktail antigen approaches show promise, multicentre validation across geographically diverse populations using standardized sample panels has not been conducted. Most significantly, WHO-standardized reference panels representing all eight genotypes do not yet exist, preventing harmonized comparison of assay performance across manufacturers and regulatory jurisdictions [4, 39]

### Clinical and pathophysiological mechanisms

The correlations between ORF2 genotype and clinical phenotype, GT1 pregnancy mortality, GT3 chronicity and neurological complications, and GT4 accelerated cirrhosis remain observational rather than mechanistically established. Several unanswered questions include whether GT1-specific ORF2 epitopes directly trigger the exaggerated inflammatory responses underlying fulminant hepatitis in pregnant women or whether placental tropism mediated by genotype-specific receptor binding variants accounts for the dramatically different pregnancy outcomes between GT1 and GT3/GT4. The molecular mimicry hypothesis linking GT3 ORF2 epitopes (particularly residues 578–607) to neurological complications through cross-reactive anti-ganglioside antibodies requires definitive validation through systematic epitope mapping against neural antigens. Chimeric virus experiments, swapping ORF2 between genotype backbones and single-substitution mutagenesis at key positions (e.g. 477, 497) in humanized mouse models are essential to establish whether ORF2 variation directly determines disease severity or merely serves as a marker of broader genomic differences between genotypes. Furthermore, the ORF2g isoform’s role in extrahepatic pathogenesis, particularly whether ORF2g-antibody immune complex deposition is a genotype-dependent phenomenon contributing differentially to glomerulonephritis and other immune-mediated complications, warrants comparative studies across GT1, GT3 and GT4 infections [4, 12].

### Methodological and technical challenges

Robust culture systems exist only for GT3 and select GT4 strains; CRISPR screening to identify host restriction factors for GT1, GT2, GT7 and GT8 is an urgent priority. GT2 is represented by a single complete genome, and sequences for GT5-GT8 remain sparse. Metagenomic recovery from archived historical samples and targeted wildlife surveillance, combined with international data-sharing mandates, are needed to enable structural, evolutionary and functional analyses across all eight genotypes [27].

Several computational and structural approaches could accelerate progress against these constraints, although we note at the outset that none has yet been applied systematically to HEV ORF2 in the published literature and the following is, therefore, a research agenda rather than a description of existing work. Deep-learning structure prediction has transformed structural biology more broadly, with AlphaFold2 achieving accuracy competitive with experimental structures for many targets and AlphaFold3 extending prediction to biomolecular complexes [92, 93]. Applied to HEV, such methods could in principle generate models of the ORF2 protomer and of P domain dimers for GT5, GT6, GT7 and GT8, for which no experimental structure exists, allowing genotype-variable residues to be mapped onto a three-dimensional surface and candidate conformational epitopes to be prioritized before crystallographic work is attempted. The reliability of such models for the specific purpose of epitope prediction would need to be established, since prediction confidence is typically lowest in the flexible surface loops that carry neutralizing epitopes. Computational epitope prediction and immunogen optimization face a more fundamental constraint for HEV: these methods depend on training data derived from monoclonal antibody escape and structural studies, and for HEV such data are confined to a small number of antibodies and genotypes, so generating the underlying experimental data is a prerequisite rather than an alternative to computational design. Cryo-electron microscopy has already been applied to HEV capsids [49] and remains the most promising route to the structures that are still missing, in particular the quasi-enveloped virion and antibody–capsid complexes that have resisted crystallization. Deep mutational scanning, in which all substitutions across a domain are assayed in parallel for effects on folding, receptor binding and antibody escape, has produced complete antibody-escape maps for influenza haemagglutinin and the SARS-CoV-2 receptor-binding domain [94, 95]; an equivalent experiment on the HEV P domain would convert the current handful of individually characterized positions into a comprehensive map of the mutational landscape, though it would first require an HEV culture or display system able to support library-scale screening, which is itself constrained by the limited culture systems described above. These approaches could offer a route to narrowing the structural and antigenic data gap for the understudied genotypes without waiting for isolates that may take years to obtain, but their outputs are hypotheses: predicted structures and epitopes require experimental validation by binding and neutralization assays before informing vaccine or diagnostic development.

### Prioritized research agenda

Addressing ORF2-related challenges requires a coordinated, multi-disciplinary research agenda with clear prioritization. Immediate priorities (1–3 years) include: (i) high-resolution structural characterization of GT4, GT7 and GT8 ORF2 proteins using cryo-electron microscopy to identify conserved epitope geometries for structure-guided vaccine design; (ii) full epitope mapping across all eight genotypes using monoclonal antibody panels and peptide arrays to define minimal conserved neutralizing epitope sets; (iii) development and validation of pan-genotype diagnostic antigens incorporating conserved epitopes from GT1, GT3, GT4, GT7 and GT8 in multicentre clinical studies and (iv) systematic analysis of ORF2 sequences from chronic infections to identify escape mutation hotspots informing therapeutic antibody cocktail design.

Medium-term priorities (3–5 years) should focus on: (i) clinical trials of next-generation vaccines employing consensus sequences, multivalent formulations or epitope-focused nanoparticle platforms in GT1-endemic and GT3-endemic regions with genotype-specific efficacy endpoints; (ii) mechanistic studies linking specific ORF2 variants to pregnancy-associated mortality, chronic infection susceptibility and neurological complications using humanized mouse models and placental explant systems; (iii) development of broadly neutralizing therapeutic monoclonal antibody cocktails with demonstrated activity against GT1-8 and resistance to escape mutations and (iv) establishment of WHO-coordinated international reference panels representing all genotypes for diagnostic assay standardization.

Long-term objectives (5–10 years) encompass: (i) achieving universal vaccine coverage through implementation of pan-genotype vaccines in both endemic regions (addressing GT1/GT2) and high-income countries (addressing GT3/GT4/GT7/GT8); (ii) integration of HEV screening into blood and organ donation protocols worldwide using validated pan-genotype molecular and serological assays; (iii) therapeutic antibody availability for high-risk populations, including immunocompromised patients and pregnant women in endemic regions and (iv) thorough surveillance systems for emerging HEV genotypes and variants enabling rapid public health response.

## Conclusions and future perspectives

This review demonstrates that ORF2 genetic and antigenic variation has functional consequences extending well beyond taxonomy. The 15–25% nucleotide divergence between genotypes translates into altered neutralizing epitope structures and genotype-specific immunopathological signatures, with positively selected sites concentrated in the P2 subdomain (residues 534–606) where antibody-mediated pressure is greatest. The coexistence of three functionally distinct isoforms (ORF2i, ORF2g, ORF2c) adds further complexity that must be integrated into vaccine and therapeutic design. Current control tools show genotype-dependent performance gaps: the GT1-derived Hecolin® vaccine demonstrates 2–4-fold reduced cross-neutralization against GT3, and commercial serological assays exhibit 12–30% false-negative rates for heterologous genotypes. Meanwhile, genotype-specific clinical phenotypes, GT1 pregnancy mortality, GT3 chronicity and neurological complications, GT4 accelerated fibrosis, point to ORF2 variation influencing pathogenesis through mechanisms that remain experimentally unestablished.

Translating these insights into clinical impact will require overcoming regulatory, economic and implementation barriers simultaneously. Pan-genotype vaccine licensure may necessitate immunological bridging studies demonstrating equivalent neutralizing antibody titres across genotypes, given the limited clinical trial infrastructure in GT1-endemic regions. For diagnostics, WHO-coordinated reference panels representing all eight genotypes are needed to harmonize assay evaluation, and genotype-specific performance standards should be mandated for manufacturers. Clinician education in GT3-endemic regions, where HEV remains under-recognized as a locally acquired pathogen, is equally important for ensuring appropriate testing and timely diagnosis.

Progress toward pan-genotype HEV control is achievable, but the near-complete absence of structural, immunological and clinical data for GT2, GT5, GT6 and GT8, combined with unresolved genotype-phenotype mechanisms and the absence of Hecolin® efficacy trials outside China, represent gaps that cannot be dismissed as minor refinements. The emergence of GT7 and GT8 with documented human infections underscores that HEV’s genotypic diversity is not static, and control strategies must accommodate genotypes not yet fully characterized. A deliberate shift from genotype-centric to pan-genotype thinking, guided by the prioritized research agenda outlined in Section 6.7, is necessary across vaccine design, diagnostic development and clinical management. ORF2, as the molecular interface between virus and host immunity, will remain central to these efforts, and a genotype-inclusive understanding of its variation is indispensable for next-generation interventions capable of controlling HEV globally.

## Abbreviations

ARM: Arginine-Rich Motif
GBS: Guillain–Barré syndrome
GT: genotype
HEV: hepatitis E virus
HSPG: Heparan sulphate proteoglycan
HSPG: Heparan sulphate proteoglycan
M: middle domain
NA: neuralgic amyotrophy
P: protruding domain
S: shell domain
SOT: solid organ transplant
VLP: virus-like particle
